# Structural Features and Potent Antidepressant Effects of Total Sterols and β-sitosterol Extracted from *Sargassum horneri*

**DOI:** 10.3390/md14070123

**Published:** 2016-06-28

**Authors:** Donghai Zhao, Lianwen Zheng, Ling Qi, Shuran Wang, Liping Guan, Yanan Xia, Jianhui Cai

**Affiliations:** 1 The Basic Medical College, Jilin Medical University, Jilin 132013, China; qiling1718@163.com (L.Q.); shuranwang@163.com (S.W.); 2 The Second Hospital, Jilin University, Changchun 130041, China; davezheng@sohu.com; 3 Food and Pharmacy College, Zhejiang Ocean University, Zhoushan 316022, China; glp730@163.com (L.G.); 18368081369@163.com (Y.X.)

**Keywords:** *Sargassum horneri*, sterols, β-sitosterol, FST, TST, monoamine neurotransmitters

## Abstract

The purified total sterols and β-sitosterol extracted from *Sargassum horneri* were evaluated for their antidepressant-like activity using the forced swim test (FST) and tail suspension test (TST) in mice. Total sterols and β-sitosterol significantly reduced the immobility time in the FST and TST. Total sterols were administered orally for 7 days at doses of 50, 100, and 200 mg/kg, and β-sitosterol was administered intraperitoneally at doses of 10, 20, and 30 mg/kg. β-sitosterol had no effect on locomotor activity in the open field test. In addition, total sterols and β-sitosterol significantly increased NE, 5-HT, and the metabolite 5-HIAA in the mouse brain, suggesting that the antidepressant-like activity may be mediated through these neurotransmitters.

## 1. Introduction

Depression is one of the most prevalent psychopathologies. Depression is characterized by a decrease in the ability to experience pleasure, lowered mood, and reduced interest. It is a chronic and disabling mental illness that causes high morbidity and mortality [1]. The World Health Organization estimated that 350 million people suffer from depression worldwide and predicted that depression will be the second leading cause of disability worldwide by 2020 [2,3]. The main biochemical causes of depression are metabolic disorders of monoamine neurotransmitters that are involved in noradrenaline (NE), serotonin (5-HT), and dopamine (DA) signaling. These neurotransmitters play important roles in mediating behavioral activity induced by antidepressant drugs [4,5]. Brain-derived neurotrophic factors (BNDF) and γ-aminobutyric acid (GABA) are believed to be associated with depressive disorders [6,7]. The function of the hypothalamic-pituitary-adrenal (HPA) axis was impaired in many depressed patients [8]. In addition, a number of evidences confirm the active role played by the glutamate (l-Glu) neuroreceptor system on the pharmacological therapeutic mechanisms of antidepressants [9]. *N*-methyl-*d*-aspartate receptors (NMDARs) or α-amino-3-hydroxy-5-methyl-4-isoxazole propionic acid l-Glu receptors (AMPARs) are major targets of the various chronic depressive disorders [10,11].

Steroids are biological signaling molecules with profound chemical, clinical, and scientific significance. Steroids have diverse biological actions that are mediated through different functional groups surrounding a rigid tetracyclic core [12]. In recent years, steroids have displayed antidepressant effects. Han et al. [13] demonstrated that steroids could significantly reduce the immobility time of mice during the forced swim test (FST), indicating an antidepressant activity. We previously showed that fucosterol, a sterol compound of *Sargassum fusiforme*, significantly reduced the immobility time of mice during the FST and the tail suspension test (TST) compared with control mice at doses of 10, 20, 30, and 40 mg/kg, indicating an antidepressant-like effect [14].

*Sargassum* is a large genus with more than 150 species, including *Sargassum horneri*, a brown seaweed found in the Northwestern Pacific Ocean and the adjoining seas of Korea, Japan, and China. Total steroid and β-sitosterol extracts from *S. horneri* have been used to treat scrofula, gall, goiter, and edema [15,16,17,18,19,20,21]. However, the purification and characterization of these extracts and their antidepressant effects have not been well studied. In this study, we have used two classic animal behavioral despair tests—the FST and TST—to evaluate the antidepressant activity of the total steroids and β-sitosterol extracts of *S. horneri* in mice. Levels of the main monoamine neurotransmitters and their metabolites were also measured in the mice brain. Our findings may be useful for the development of novel drugs and food to treat depression.

## 2. Results and Discussion

### 2.1. Chemical Analysis

Total sterol and β-sitosterol extracts displayed a positive Liebermann-Burchard reaction, which characterizes sterol materials. The chemical structure of β-sitosterol was characterized by IR, ^1^H NMR, ^13^C NMR, and mass spectroscopy. The IR spectra of β-sitosterol revealed –OH stretching (3397 cm^−1^) bands, –CH_3_ stretching (2932 cm^−1^), and –C=C– stretching (1644 cm^−1^). In the ^1^H-NMR spectra of β-sitosterol, –OH protons were observed as broad bands at 10.2 ppm and two –CH_3_ protons were displayed as single bands at 0.78 ppm (3H, s, H-18) and 1. 08 ppm (3H, s, H-19). =CH and –CH– protons were shown as a broad band at 5.41 (1H, br d, H-6) and as multiple peaks at 3.60 (1H, m, H-3), which is characteristic of hydroxyl sterols of the mother nucleus for Δ^5^-3β–. In the ^13^C-NMR spectra of β-sitosterol, three –C– groups were seen at 141.21 ppm (C-5), 44.35 ppm (C-13), and 37.43 ppm (C-10); nine –CH– groups were observed at 120.97 ppm (C-6), 72.06 ppm (C-3), 58.34 ppm (C-17), 56.76 ppm (C-14), 49.98 ppm (C-9), 47.13 ppm (C-24), 36.35 ppm (C-20), 32.01 ppm (C-8), and 31.88 ppm (C-25); eleven –CH_2_– groups were observed at 41.98 ppm (C-4), 37.21 ppm (C-12), 34.09 ppm (C-22), 32.12 ppm (C-2), 30.46 ppm (C-1), 29.89 ppm (C-7), 28.02 ppm (C-23), 27.74 ppm (C-15), 27.28 ppm (C-16), 25.90 ppm (C-29), and 23.71 ppm (C-11); and six –CH_3_– groups were observed at 22.80 ppm (C-19), 21.35 ppm (C-18), 21.07 ppm (C-21), 19.89 ppm (C-26), 18.78 ppm (C-27), and 12.77 ppm (C-29).

### 2.2. Effects of Total Sterol Extract and β-sitosterol on Immobility Time in the FST and TST

The antidepressant-like activity of total sterols and fluoxetine on the immobility time in these tests are presented in Table 1. Total sterols significantly reduced the immobility time in the FST and TST at doses of 50, 100, and 200 mg/kg for 7 days following oral administration (except 50 mg/kg in the TST). Total sterols had a similar effect to the drug fluoxetine (20 mg/kg) at a dose of 200 mg/kg, which induced the greatest reduction in the immobility time compared with the control group (*P* < 0.001). To better understand the antidepressant effects of total sterols and fluoxetine, we calculated the percentage decrease in immobility duration (% DID) using the formula % DID = [(*A* − *B*)/*A*] × 100, where *A* is the duration of immobility (s) in the control group, and *B* is the duration of immobility (s) in the sterol-treated group. The duration of immobility in the FST was reduced and the % DID increased by three doses of total sterols in the FST. The same effects were observed for two doses of total sterols in the TST (Table 1). The % DID values were as follows: FST: 30.44% (50 mg/kg), 42.90% (100 mg/kg) and 54.21% (200 mg/kg); TST: 49.57% (100 mg/kg), and 52.37% (200 mg/kg).

**Figure 1:**
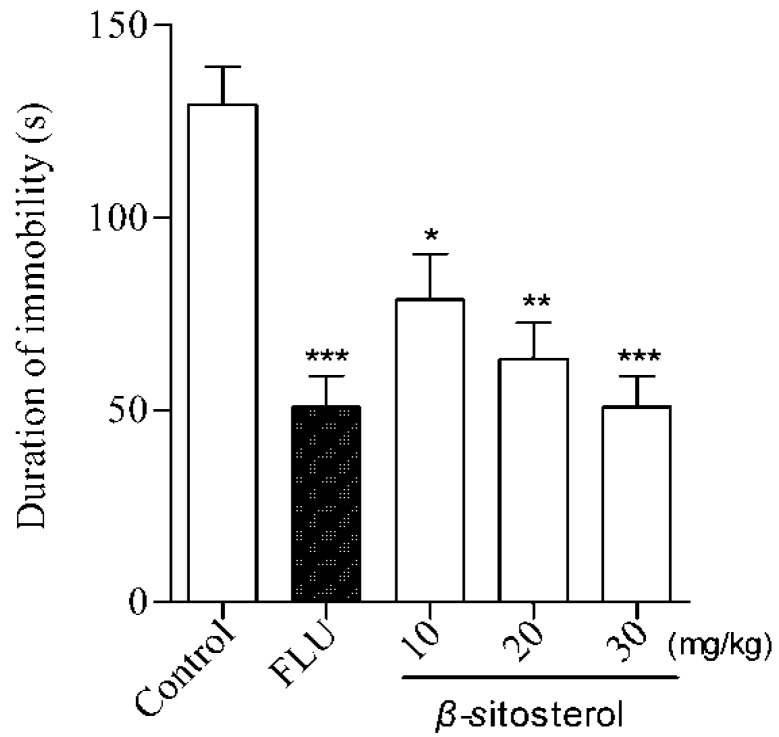
Effects of β-sitosterol and the positive control fluoxetine (FLU) on the immobility time in the FST in mice. Data are expressed as the mean ± SEM (*n* = 8). * *P* < 0.05, ** *P* < 0.01, *** *P* < 0.001 compared with vehicle control.

**Figure 2:**
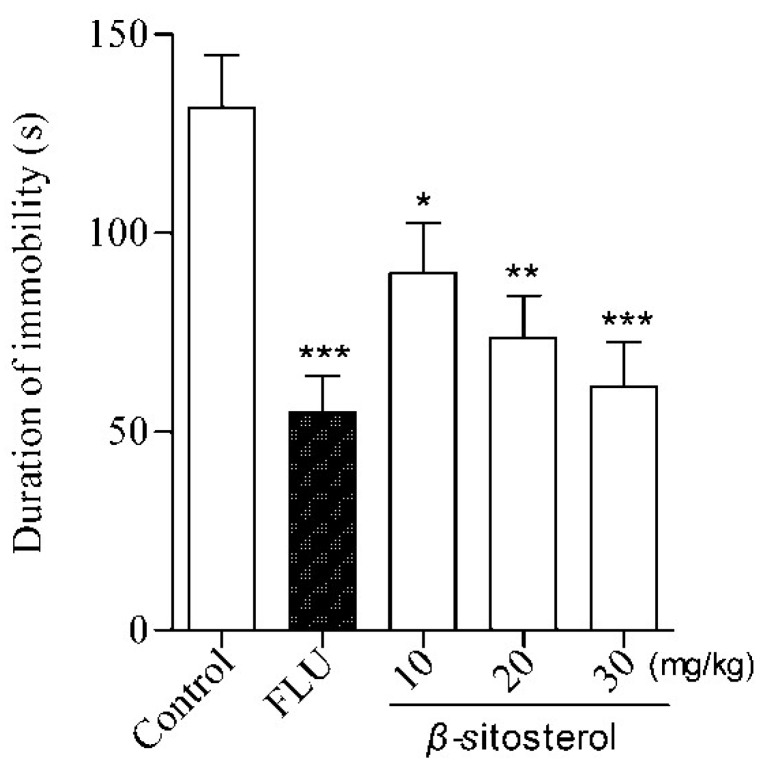
Effects of β-sitosterol and the positive control fluoxetine (FLU) on the immobility time in the TST in mice. Data are expressed as the mean ± SEM (*n* = 8). * *P* < 0.05, ** *P* < 0.01, *** *P* < 0.001 compared with vehicle control.

β-sitosterol treatment significantly reduced the immobility time at three doses (10, 20, and 30 mg/kg) in the FST and TST (Table 1 and Figure 1 and Figure 2), indicating an antidepressant effect. This effect was similar to the positive control fluoxetine (20 mg/kg) at a dose of 30 mg/kg, where the strongest effect was observed compared with the control group (*P* < 0.001). The same effects were observed for three doses of β-sitosterol in the TST (Table 1). The % DID values were as follows: FST: 39.27% (10 mg/kg), 51.23% (20 mg/kg), and 57.48% (30 mg/kg); TST: 31.63% (10 mg/kg), 43.95% (20 mg/kg), and 53.38% (30 mg/kg). These results indicate that β-sitosterol has a significant antidepressant activity in mice during the FST and TST. Furthermore, β-sitosterol exhibited the antidepressant effect in a dose-dependent manner. 

The key issue in screening for new antidepressant-like drugs is to establish a valid paradigm that is capable of accurately identifying various antidepressant treatments [22,23]. Behavioral stress paradigms are most commonly used to assess antidepressants because of their ease of use, reliability, and specificity [24,25]. The FST and TST are accepted stress models of depression. They induce a state of despair and have good reliability and predictive validity in mice. Mice are restricted and cannot escape during the FST and TST, inducing a characteristic immobility behavior. This immobility has been hypothesized to reflect behavioral despair, which may reflect depressive disorders in humans. In addition, many antidepressant drugs can reduce immobility time in rodents [26,27]. In this present study, the antidepressant effects of total sterols and β-sitosterol were firstly evaluated by using these two models. It was observed that total sterols and β-sitosterol significantly reduced the immobility time in these two models, and the decrease in immobility time was dose-dependent.

### 2.3. Effect on the Open-Field Test

**Figure 3:**
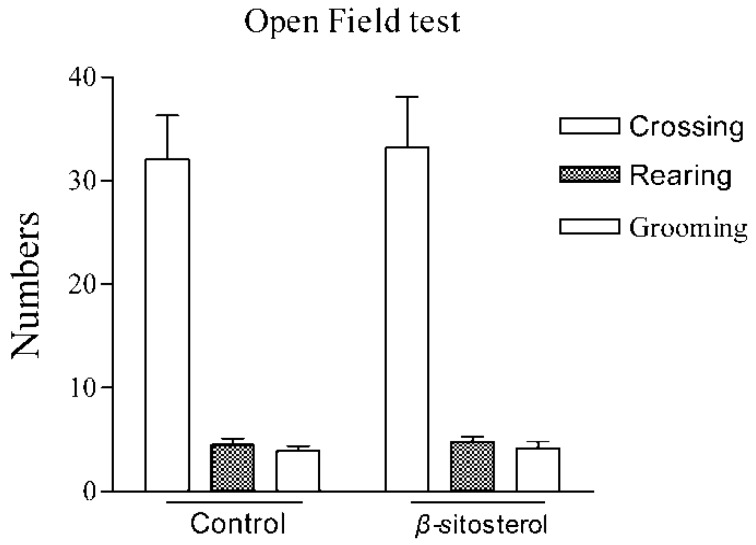
Effects of β-sitosterol on exploratory activity in the open field test. Crossing: number of line crossings; rearing: number of times observed standing on hind legs; grooming: number of modifications. Values represent the mean ± SEM *n* = 8.

In these behavioral tests, false-positive results are occasionally obtained with agents that stimulate locomotor activity [28]. Therefore, the effect of β-sitosterol on locomotor activity was evaluated using the locomotor activity test. In the open-field test, β-sitosterol treatment showed no differences compared with control animals in 5 min at the dose range used in the present study (Control: crossing = 32.61 ± 13.5, rearing = 7.73 ± 8.9, grooming = 6.32 ± 9.4; β-sitosterol: crossing = 33.99 ± 12.8, rearing = 8.37 ± 9.9, grooming = 7.08 ± 8.7). The administration of β-sitosterol in the FST did not cause any significant change in the number of crossing, rearing, and grooming in the open field test in the number of (Figure 3), demonstrating that the antidepressant-like effect of β-sitosterol in the FST and TST was not caused by altered locomotor behavior. A variety of antidepressant drugs are known to reduce the immobility time in the FST and TST. However, drugs that affect motor function may give false-positive or -negative results in the FST and TST. Psychomotor stimulants enhance locomotor activity, which reduces the immobility time [29,30].

### 2.4. Effects of Total Sterols and β-sitosterol on Monoamine Neurotransmitter Levels

The levels of monoamine neurotransmitters and their metabolites detected in mice brain are summarized in Table 2. In the present study, total sterols and β-sitosterol did not change DA levels, but significantly increased 5-HT and NE levels at the highest doses during the FST in mice brain, similar to the positive control drug fluoxetine. In addition, total sterols and β-sitosterol significantly increased 5-HIAA levels, indicating a reduced 5-HT metabolism. Enhancing 5-HT and/or NE neurotransmission is currently the most efficacious treatment for depression [31,32,33]. Our findings suggest that the antidepressant-like effect of total sterols and β-sitosterol are likely mediated through increased 5-HT and NE levels in the central nervous system. Some studies have also shown the adaptogenic effect of the plant extract via normalization of the various stress parameters and monoaminergic levels, which may provide a clue that the extract is bringing their possible antidepressant effect through restoration of normal monoaminergic neurotransmitters [34,35]. Dysregulation of the central nervous system neurotransmitters 5-HT, NE, and DA are thought to play a role in the pathogenesis of depression. The majority of studies have focused on the 5-HT and NE systems. A metabolic disorder of monoamine neurotransmitters is believed to be the main biochemical cause of depression, and increasing the levels of monoamine neurotransmitters in the central nervous system can alleviate the symptoms of depression [36,37,38]. Reduced concentrations of 5-hydroxyindol-eacetic acid (5-HIAA) (the major metabolite of 5-HT) have been observed in the brains of patients and animals experiencing stress and depression, indicating a dysfunction of the serotonergic system [39,40].

We investigated the effect of total sterols and β-sitosterol extracted from *Sargassum horneri* on depressive behavior in the FST and TST because these have been widely used as a herbal medicine in the past.

## 3. Experimental Section

### 3.1. Materials and Agents

*Sargassum horneri* was collected in Donghai, Zhejiang Province (China). A positive control: fluoxetine-HCl (Aldrich Chemical Corporation, Shanghai, China, purity > 99%) was included. Reference standards for simultaneous determination of 5-HT, NE, DA, and 5-HIAA were purchased from Sigma (Shanghai, China, purity > 99%). Melting points were determined in open capillary tubes. IR spectra were recorded (in KBr) on a FT-IR1730 (Bruker, Karlsruhe, Germany), and ^1^H-NMR and ^13^C-NMR spectra were measured on an AV-500 (Bruker, Rheinstetten, Switzerland). High-resolution mass spectra were measured on an MALDI-TOF/TOF mass spectrometer (Bruker Daltonik, Germany). The major chemicals were purchased from Aldrich Chemical Corporation (Shanghai, China). All other chemicals were of analytical grade.

### 3.2. Animals

Male ICR (Institute of Cancer Research) mice (20 ± 2 g) obtained by the Laboratory of Animal Research, College of Pharmacy, Zhejiang Academy of Medical Sciences. Male KunMing mice (20 ± 2 g) obtained by the Laboratory of Animal Research, Jilin University. Mice were acclimatized for 1 week before being used for the experiment. Before and during the experiment, the mice were housed under controlled environmental conditions of temperature (23 ± 2 °C), humidity 40%–60%, and maintained under a 12 h:12 h light-dark cycle (lights on at 8:00 A.M.) with free access to food and tap water. The procedures in this study were performed in accordance with the National Institute of Health Guide for the Care and Use of Laboratory Animals and approved by the Ethics Committee of our Institution. All efforts were made to minimize animals suffering and to reduce the number of animals used in the experiments.

### 3.3. Isolation and Purification of Total Sterols and β-sitosterol

**Figure 4:**
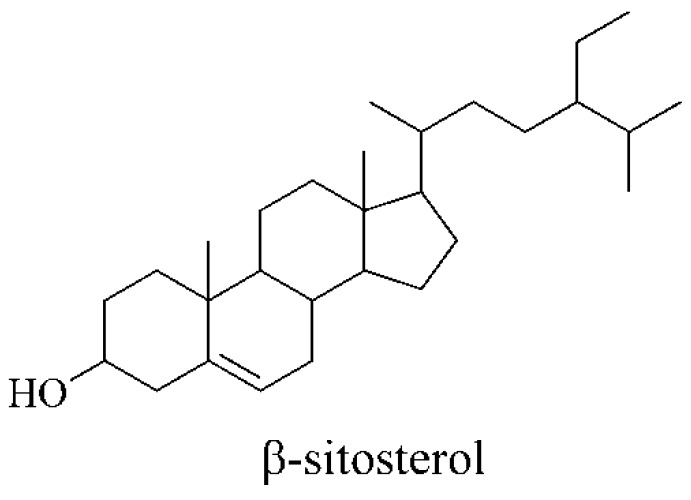
Chemical structure of β-sitosterol.

The total sterols and β-sitosterol were extracted from *Sargassum horneri* with root cut off according to the procedures described in previous study [41]. Briefly, the air-dried *S.* *horneri* were extracted with three-time 95% ethanol under reflux and repeated for 3 h thrice. All ethanol-extracts were combined, filtrated, and concentrated under vacuum to afford the crude material. The mixture was added and dissolved with water, and extracted sequentially with *n*-hexane, acetic ether, and *n*-butanol. The *n*-hexane fraction provided to the total steroids. Then, *n*-hexane fraction was subjected to a silica gel column eluted with n-hexane-acetic ether, and acetic ether-MeOH affording ten sub-fractions (sfr): sfr.1 to sfr.10. Subfraction 6 was subjected to isocratic semi-preparation HPLC using with n-hexane-acetic ether afford the white solid β-sitosterol (Figure 4). The melting point and spectral data of β-sitosterol are given as below. Mp. 136.7–138.4 °C; IR (KBr) cm^−1^: 3397, 2932, 1644, 1470, 1341, 1050; ^1^H-NMR (CDCl_3,_ 500 MHz): *δ* 0.78 (3H, s, H-18), 1.02 (3H, d, H-21), 1.08 (3H, s, H-19), 1.61 (3H, t, H-29), 2.18 (1H, m, H-25), 3.60 (1H, m, H-3), 5.41 (1H, br d, *J* = 5.12 Hz, H-6), 10.2 (1H, s, –OH); ^13^C-NMR (CDCl_3_, 100 MHz): δ 141.21, 120.97, 72.06, 58.34, 56.76, 49.98, 47.13, 44.35, 41.98, 37.43, 37.21, 36.35, 34.09, 32.12, 32.01, 31.88, 30.46, 29.89, 28.02, 27.24, 27.28, 25.90, 23.71, 22.80, 21.35, 21.07, 19.89, 18.78, 12.77; ESI-HRMS calcd. for C_29_H_50_O^+^ ([M + H]^+^): 415.3862; found: 415.3858. The comprehensive analysis of the above data consistent with that reported in the literature [42].

### 3.4. Drug Treatment

β-sitosterol was dissolved in DMSO, the total sterols were dissolved 0.2% carboxyemethyl cellulose sodium (CMC-Na), and other drugs were dissolved in isotonic saline solution (NaCl 0.9%) immediately before use. Vehicle solvent served as a negative control, while fluoxetine served as a positive control. The total sterols were administered by suspension via gastric intubation route with 0.2 mL/20 g of mice. The same dose was administered every day via gastric administration once a day and successively for 7 days before in the FST and TST. β-sitosterol and all drugs were administered by intraperitoneal (ip) route 30 min before in the FST, TST, or open-field test, and the volume of administration for vehicle and drug solutions was 0.1 mL/20 g of mice.

### 3.5. The Forced Swim Test

Male ICR mice (20 ± 2 g) were used in the forced swim test under standard conditions with free access to food and water. Mice were randomly divided into four groups (8 mice per group were used) for the forced swim test (FST): β-sitosterol (10, 20, and 30 mg/kg), total sterols (50, 100, and 200 mg/kg), fluoxetine (20 mg/kg), or distilled water. We used 80 male mice. On the test day, mice were dropped one at a time into a plexiglass cylinder (height 25 cm, diameter 10 cm) containing 10 cm of water at 20 ± 3 °C. On this day, mice were assigned into different groups (*n* = 8 for each group). The vehicle or test drugs were administered 30 min before a test session acute ip injection. Then, mice were dropped individually into the plexiglass cylinder and left in the water for 6 min. After the first 2 min of the initial vigorous struggling, the animals were immobile. The duration of immobility was recorded during the last 4 min of the 6 min test. All test swim sessions were recorded by a video camera positioned directly above the cylinder. Two competent observers, who were unaware of the treatment each mouse had received, scored the videotapes. Immobility period was regarded as the time spent by the mouse floating in the water without struggling and making only those movements necessary to keep its head above the water. Following swimming sessions, they were then towel dried and returned to their housing condition. The animals were used only once in this test. All FSTs were performed between 10:00 A.M. and 14:00 P.M. [43].

### 3.6. The Tail Suspension Test

Local breed, male ICR mice (20 ± 2 g) were used in the FST under standard conditions with free access to food and water. Mice were randomly divided into four groups (8 mice per group were used) for the tail suspension test (TST): β-sitosterol (10, 20, and 30 mg/kg), total sterols (50, 100, and 200 mg/kg), fluoxetine (20 mg/kg), or distilled water. We used 80 male mice. Briefly, the vehicle or test drugs were administered 30 min before a test session acute ip injection. Then, mice were individually suspended by tail with clamp (2 cm from the tip of the end) in a box (25 cm × 25 cm × 30 cm) with the head 5 cm to the bottom. Testing was carried out in a darkened room with minimal background noise. All animals were suspended for total 6 min, and the duration of immobility was observed and measured during the final 4-min interval of the test. All test sessions were recorded by a video camera positioned directly above the box. Two competent observers blind to treatment scored the videotapes. Mice consider immobile only when they hung passively and completely motionless. The animals were used only once in this test. All TSTs were performed between 11:00 A.M. and 14:00 P.M. [44].

### 3.7. The Open-Field Test

The open-field test was carried out in mice according to the method for previously reported in the literature with slight modifications. Mice were placed individually in the center of the open-field apparatus, and the locomotor activity was assessed immediately before the FST [28]. The open-field apparatus was a square, 40 cm on every side, which was demarcated into 16 equal areas. The score locomotion (number of line crossings within 5 min), rearing frequencies (number of times an animal stood on its hind legs), and grooming (number of modifications) were recorded. In this experiment, mice received the same drugs and doses as those used when measuring immobility. The open-field apparatus was washed with a deodorant solution and dried before each behavioral test to eliminate possible odor clues left by previous subjects. Experiments were performed in a dark room, and the apparatus was illuminated by a 60-W bulb positioned 1 m above the center of the circle. 

### 3.8. The Sample Preparation

The doses of the total sterols (200 mg/kg) and the doses of β-sitosterol and fluoxetine (20 mg/kg) were employed to test the effect on monoamine neurotransmitter concentrations in the brain of mice. Mice were randomly divided into five groups (10 mice per group were used); we used 50 male mice. Normal vehicle, stress vehicle, total sterols, β-sitosterol, and fluoxetine were given orally daily for 7 days. On the last day, the drugs were given 1 h prior to the test. At the end of the experiment, the mice were immediately sacrificed by cervical dislocation, the brain tissue was quickly removed, and rapidly frozen and stored at −80 °C until they were processed for biochemical estimations.

### 3.9. HPLC Condition and Test

The brain tissues were sonicated in 0.1 M NaH_2_PO_4_ aqueous solution including 0.85 mM OSA, 0.5 mM Na_2_·EDTA (ethylenediamine tetraacetic acid disodium), and centrifuged at 13,000× *g* for 15 min at 4 °C. Then, 5-HT, NE, DA, 5-HIAA, and DHBA were assayed by HPLC-ECD. Equipment used was as follows: Shimadzu LC-10ATVP HPLC system, Shimadzu L-ECD-6A electrochemical detector, N2000 HPLC workstation software, and Hypersil ODS C18 Column 4.6 × 150 mm (5 μm). The mobile phase consisted of 0.1 M NaH_2_PO_4_ aqueous solution including 0.85 mM OSA, 0.5 mM Na_2_·EDTA, and 11% methanol adjusted to pH 3.4 with phosphate acid and filtered through 0.45 μM pore size filter. External standard curves were used to quantify the amounts of 5-HT, NE, DA, and 5-HIAA in each sample calculated by area under curve. The volume of injection was 20 μL. The detection limit of the assay was 20 pg/g samples. The filtrate sample was used for the quantification of 5-HT, NE, DA, and 5-HIAA by HPLC coupled with electro-chemical detection in the brain region.

### 3.10. Statistical Analysis 

Statistical analysis was performed using GraphPad Prism (GraphPad Prism 5.0, version 2.0; GraphPad Software, Inc., San Diego, CA, USA). The results are expressed as the mean ± SEM The Student’s *t*-test was used to compare the differences between the two groups, with a one-way analysis of variance (ANOVA) followed by Tukey’s multiple comparison tests. A value of *P* < 0.05 was considered significant.

## 4. Conclusions

In conclusion, we have here demonstrated that total sterols and β-sitosterol extracts from *Sargassum horneri* significantly reduce the immobility time in the FST and TST, indicating an antidepressant effect. Furthermore, we found that the antidepressant effect of total sterols and β-sitosterol is likely mediated by an increase in 5-HT and NE in the central nervous system.

## Figures and Tables

**Table 1:** Evaluation of the antidepressant-like effect of total sterols in the forced swim test (FST) and tail suspension test (TST).

Compounds	Dose (mg/kg)	Antidepressant Activity ^1^	
		Duration of Immobility (s)	DID (%) ^2^
**FST**			
Total sterols	50	91.6 ± 12.1 *	30.44
	100	75.2 ± 9.4 **	42.90
	200	60.3 ± 9.6 ***	54.21
Fluoxetine	20	62.5 ± 6.5 ***	52.54
Control	—	131.7 ± 11.3	—
β-sitosterol	10	78.7 ± 11.9 *	39.27
	20	63.2 ± 9.5 **	51.23
	30	55.1 ± 10.1 ***	57.48
Fluoxetine	20	50.8 ± 8.1 ***	60.80
Control	—	129.6 ± 9.8	—
**TST**			
Total sterols	50	99.6 ± 10.1	28.45
	100	70.2 ± 10.6 **	49.57
	200	66.3 ± 8.2 ***	52.37
Fluoxetine	20	65.5 ± 8.5 ***	52.95
Control	—	139.2 ± 12.5	—
β-sitosterol	10	89.9 ± 12.7 *	31.63
	20	73.7 ± 10.5 **	43.95
	30	61.3 ± 11.3 ***	53.38
Fluoxetine	20	54.8 ± 9.3 ***	58.33
Control	—	131.5 ± 13.3	—

^1^ Total sterols and fluoxetine were administered oral route. Values are the mean ± standard error of the mean (SEM) (*n* = 8); ^2^ % DID: percentage decrease in immobility duration. * *P* < 0.05, ** *P* < 0.01, *** *P* < 0.001 compared with the control.

**Table 2:** Effects of the FST exposure, total sterols, and β-sitosterol on brain monoamine neurotransmitter levels.

Groups	5-HT	NE	DA	5-HIAA
Normal vehicle	316.4 ± 30.6	217.6 ± 27.3	401.3 ± 39.5	273.7 ± 28.3
Stress vehicle	202.1 ± 29.3	197.8 ± 22.4	346.3 ± 41.2	132.1 ± 26.4
Total sterols	348.2 ± 29.5 ^a,d^	358.5 ± 20.2 ^a,d^	299.8 ± 39.4	271.3 ± 27.2 ^b^
β-sitosterol	387.1 ± 38.5 ^b,d^	398.2 ± 31.5 ^c,e^	327.3 ± 38.6	312.1 ± 31.3 ^a^
Fluoxetine	373.6 ± 39.4 ^b,d^	389.5 ± 33.1 ^c,e^	302.4 ± 40.1	289.2 ± 33.4 ^b^

The dose of total sterols was 200 mg/kg, the doses of β-sitosterol and fluoxetine were 20 mg/kg. Neurotransmitter levels are expressed as ng/g per brain wet weight. Data are expressed as mean ± SEM *n* = 10. Statistical analyses were conducted using one-way analysis of variance (ANOVA) followed by Tukey’s test. ^a^ *P* < 0.05, ^b^ *P* < 0.01, ^c^ *P* < 0.001 vs. stress vehicle; ^d^ *P* < 0.05, ^e^ *P* < 0.01 vs. normal vehicle.
